# Association of dietary vitamin C consumption with severe headache or migraine among adults: a cross-sectional study of NHANES 1999–2004

**DOI:** 10.3389/fnut.2024.1412031

**Published:** 2024-06-18

**Authors:** Yafang Zheng, Jing Jin, Chuanxiang Wei, Chunyuan Huang

**Affiliations:** Liaoning University of Traditional Chinese Medicine, Shenyang, Liaoning, China

**Keywords:** dietary vitamin C consumption, severe headache or migraine, antioxidant, NHANES, adults

## Abstract

**Background:**

An antioxidant-rich diet has been shown to protect against migraines in previous research. However, little has been discovered regarding the association between migraines and vitamin C (an essential dietary antioxidant). This study assessed the dietary vitamin C intake among adult migraineurs in the United States to determine if there is a correlation between migraine incidence and vitamin C consumption in adults.

**Methods:**

This cross-sectional research encompassed adults who participated in the National Health and Nutrition Examination Survey (NHANES) from 1999 to 2004, providing detailed information on their dietary vitamin C intake as well as their history of severe headaches or migraines. The study used weighted multivariable and logistic regression analyses to find an independent connection between vitamin C consumption and severe headache or migraine. Tests of interactions and subgroup analysis were conducted.

**Results:**

Among the 13,445 individuals in the sample, 20.42% had a severe headache or migraine. In fully adjusted models, dietary vitamin C consumption was substantially linked negatively with severe headache or migraine (odds ratio [OR] = 0.94, 95% confidence interval [CI] = 0.91–0.98, *p* = 0.0007). Compared to quartile 1, quartile 4 had 22% fewer odds of having a severe headache or migraine (OR = 0.78, 95% CI = 0.69–0.89, *p* = 0.0002). Subgroup analyses showed a significant difference between vitamin C intake and severe headaches or migraines by gender (*p* for interaction < 0.01).

**Conclusion:**

Reduced risk of severe headaches or migraines may be associated with increased consumption of vitamin C.

## 1 Introduction

The typical neurological ailment known as migraine generally presents as a moderate to severe throbbing headache that is unilateral in nature. Other frequently experienced symptoms include nausea, vomiting, photophobia, phonophobia, and various others. The two primary clinical subtypes of migraine are migraine with aura (MA) and migraine without aura (MO) (1). Globally, approximately 1 billion individuals suffer from migraines, making it the second most prevalent cause of disability overall and the primary source of impairment for women aged 15 to 49 (2, 3). Research on the population in the United States shows that about 6% of men and 18% of women get migraine headaches (4).

Notwithstanding the prevalence of migraines, their pathogenesis is complex and incompletely comprehended. As a multifactorial and polygenic disease, migraine headache may mainly result from fluctuations in serotonin concentration caused by biochemical factors, depolarization of neurons and glial cells caused by cortical spreading depression (CSD), and neurogenic inflammation in the trigeminovascular system (5–8). In addition, vascular dysfunction, activation of trigeminal vascular pathways, and transmission of injurious messages are key processes, in which the release of neuropeptides may trigger physiological responses such as arteriolar vasodilation. Meanwhile, oxidative stress is a major factor in the genesis of migraines. Disturbances in cellular biochemical pathways may be responsible for this stress, which in turn affects intracellular molecular structure and function (9–11).

Antioxidant intervention in migraine therapeutic therapy has long been a popular area of study. Numerous studies have shown that migraine sufferers benefit from consuming antioxidants such as vitamin C, coenzyme Q10, and curcumin, etc. (12, 13). In the respiratory chain of the mitochondria, coenzyme Q10 transports electrons. Its primary function is to shield lipids, proteins, and DNA from oxidative damage (14). Coenzyme Q10 supplementation is advised by the American Academy of Neurology as a migraine-preventative strategy (level of evidence C) (15). Curcumin, a polyphenolic molecule derived from turmeric, possesses anti-inflammatory, antioxidant, anti-protein aggregation, and analgesic properties, rendering it a viable candidate for preventing and treating migraine (16).

Both oxidized L-dehydroascorbic acid and L-ascorbic acid are forms of vitamin C, which is an important water-soluble chemical molecule. Because they lack L-gulonolactone oxidase, humans can only obtain vitamin C from their diet of fruits and vegetables (17, 18). Vitamin C is known as an antioxidant because of its ability to prevent other compounds from being oxidized by providing electron donors (19). It demonstrates antioxidant properties through neutralizing reactive molecules like reactive oxygen species (ROS) and reactive nitrogen species (RNS). Apart from directly neutralizing ROS, vitamin C aids in the regeneration of other antioxidants in the organism, for instance, vitamin E. For this reason, vitamin C is a necessary redox node, especially in conjunction with glutathione (20). Vitamin C also possesses lipid peroxidation-reducing and anti-inflammatory qualities (21, 22). The brain's antioxidant defense mechanism is vulnerable due to its elevated oxygen utilization, surplus polyunsaturated fatty acids, relative scarcity of antioxidants, and the non-renewable nature of CNS cells. This renders the brain especially sensitive to oxidative stress (23–26). Antioxidants are critical to the brain's function. In brain tissue, glutathione predominates as an antioxidant, with vitamin C following (27). A significant fat-soluble antioxidant is vitamin E, which is also referred to as α-tocopherol. It is a necessary micronutrient that the body cannot produce on its own and must be obtained through food (28). In a lipid milieu, vitamin E can scavenge peroxyl radicals, stop polyunsaturated fatty acids from further oxidizing, and work in concert with glutathione or vitamin C. In addition, vitamin E may regulate cellular signaling processes through its antioxidant capacity (29–31).

As early as 1951, researchers discovered that vitamin C, as a dietary supplement, not only effectively strengthens the body's defense system, but may also help relieve the symptoms of migraine headaches (32). Nevertheless, no research has looked at the connection between migraines in the general population and dietary vitamin C consumption. To fill this gap, we used NHANES data to study the link between dietary vitamin C consumption and severe headaches or migraines in adults.

## 2 Materials and methods

### 2.1 Study population

**Figure 1:**
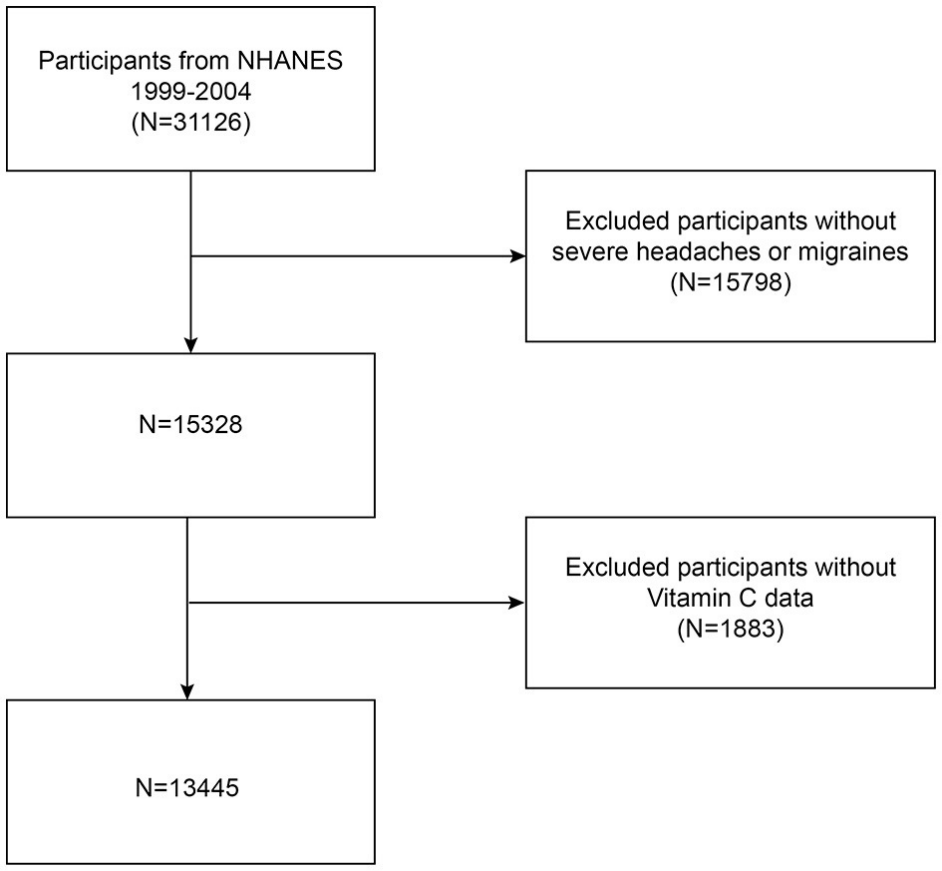
Flowchart for including and excluding participants for analysis.

Our data comes from NHANES, a National Center for Health Statistics (NCHS) study of the American people that is conducted continuously. It provides an abundance of information regarding the diet and general health of Americans. NHANES uses a well-represented sample since it is done every 2 years utilizing a stratified, multistage probability sampling process. The NCHS Ethics Committee authorized the study methods and the informed consent forms before data collection ever started. All information is publicly accessible at https://www.cdc.gov/nchs/nhanes/. Since the 1999–2004 NHANES survey cycle was the only one with a question on severe headaches or migraines, it was used for this study. A total of 31,126 eligible participants were initially recruited, and after excluding 15,798 participants with absent data on severe headache or migraine and 1,883 participants with incomplete data on dietary vitamin C intake, every person that remained was an adult who was at least 20 years old. Ultimately, 13,445 individuals in all took part in the research. The process of selecting the research population is shown in Figure 1.

### 2.2 Evaluating a migraine or severe headache

The “Miscellaneous Pain (MPQ)” portion of the NHANES questionnaire was used to collect data on migraines. The MPQ090 inquires, “Have you experienced severe headaches or migraines in the last 3 months?” Individuals who said “yes” were thought to be suffering from migraines. The majority of patients with severe headaches most likely experienced migraines, even though NHANES was unable to provide pertinent information on headache or migraine type. The 2004 U.S. Migraine Prevalence and Prevention Study data analysis was used by American Migraine Prevalence and Prevention (AMPP) to publish the overall prevalence of migraine, probable migraine (PM), and additional severe headaches. The International Classification of Headache Disorders-2 identified 11.8% of cases as migraine, 4.6% as PM, and 17.4% as “severe headache” among the 64.9% of participants who responded. Only 1% of cases were categorized as “other severe headaches” (33).

### 2.3 Dietary assessment

Dietary vitamin C consumption in milligrams based on the NHANES Dietary Interview-Total Nutrient Intake data. Two interviews lasting 24 h each were used to collect the data for this paper. A dietary interviewer performed the first interview at the Mobile Examination Center (MEC), and then after 3 to 10 days, the phone interview was used for the second one. Information from the Dietary Recall Interview was gathered in two phases: in 2003 and 2004, the USDA's Automated Multiple Pass Method (AMPM) was used, and a technique for computer-assisted nutrition interviews was verified in 1999 and 2002. In the NHANES 1999–2002 cycle, only dietary data from the first 24-h dietary recall interview was available, while in 2003–2004 there were two dietary recall interviews. To maintain consistency with the 1999–2002 period, this study included only the dietary data from the 1^st^ day of the 2003–2004 period.

### 2.4 Covariables

In this research, age, gender, race, education level, body mass index (BMI), physical activity, marital status, smoking, diabetes, hypertension, stroke, coronary heart disease, alcohol consumption, and Family Poverty Income Ratio (Family PIR) were among the covariates that were looked at. Four categories were assigned to race: non-Hispanic white, non-Hispanic black, Mexican American, and others. Classifications for education levels include <high school, high school, and >high school. The following BMI ranges were used: ≤ 25 kg/m^2^, 26–30 kg/m^2^, and >30 kg/m^2^. Physical activity was categorized as moderately active, not moderately active, and not able to perform activities. Marital status is classified as living alone, married, or with a partner. Cigarette smoking was classified as either never or ever, with <100 cigarettes in one's lifespan considered never smoking and ≥100 cigarettes in one's lifespan considered ever smoking. Data on diabetes mellitus, hypertension, stroke, and coronary heart disease were derived from self-reports on questionnaires. Alcohol consumption was the frequency of alcohol consumption by participants in the past 12 months.

### 2.5 Statistical analyses

For all statistical analyses, R (version 4.1.3) and EmpowerStats (version 2.0) were used, and statistical significance was defined as *P* < 0.05. The phrase “continuous variable” refers to mean ± standard deviation (SD), whereas “categorical variable” refers to percentages. Comparing the migraine group to the no-migraine group was done using a weighted chi-square test (for categorical variables) or a weighted *t*-test (for continuous variables). We employed a logarithmic transformation to convert the skewed distribution of vitamin C consumption into a normal distribution since the intake was skewed. Odds ratios (OR) and 95% confidence intervals (CI) between transformed vitamin C consumption and migraine were derived through multiple logistic regression analysis. Three models were produced by the multivariate tests: no variable adjustments are included in Model 1, age, gender, and race adjustments are included in Model 2, and all of the Table 1 variables are adjusted in Model 3. The non-linear correlation between transformed vitamin C intake and severe headaches or migraines was evaluated using smooth curve fitting. In order to test for heterogeneity in the associations across subgroups, subgroup analyses were also performed by adding interaction terms and stratifying the variables according to age, sex, race, and BMI. Missing values are entered by the median of continuous variables or by the multitude of categorical variables for the existing situation of these variables.

**Table 1:** The 1999–2004 National Health and Nutrition Examination Survey (NHANES) included 13,445 individuals aged 20–85 years.

**Characteristics**	**Total**	**Migraine YES**	**Migraine NO**	***P*-value**
	***N*** = **13,445**	***N*** = **2,745**	***N*** = **10,700**	
Age (years)	49.81 ± 19.06	43.62 ± 16.31	51.39 ± 19.39	<0.001
**Gender**, ***n*** **(%)**				<0.001
Male	6,371 (47.39%)	881 (32.09%)	5,490 (51.31%)	
Female	7,074 (52.61%)	1,864 (67.91%)	5,210 (48.69%)	
**Race**, ***n*** **(%)**				<0.001
Non-Hispanic white	6,783 (50.45%)	1,239 (45.14%)	5,544 (51.81%)	
Non-Hispanic black	2,555 (19.00%)	569 (20.73%)	1,986 (18.56%)	
Mexican American	3,028 (22.52%)	675 (24.59%)	2,353 (21.99%)	
Other race	1,079 (8.03%)	262 (9.54%)	817 (7.64%)	
**Education level**, ***n*** **(%)**				<0.001
<High school	4,351 (32.36%)	1,004 (36.58%)	3,347 (31.28%)	
High school	3,182 (23.67%)	659 (24.01%)	2,523 (23.58%)	
>High school	5,911 (43.97%)	1,082 (39.42%)	4,829 (45.14%)	
**BMI (kg/m**^2^**)**, ***n*** **(%)**				<0.001
≤ 25	4,163 (30.96%)	827 (30.13%)	3,336 (31.18%)	
26–30	5,069 (37.70%)	923 (33.62%)	4,146 (38.75%)	
>30	4,213 (31.34%)	995 (36.25%)	3,218 (30.07%)	
**Moderate activities**, ***n*** **(%)**				<0.001
Yes	5,910 (43.96%)	1,122 (40.87%)	4,788 (44.75%)	
No	7,032 (52.31%)	1,504 (54.79%)	5,528 (51.67%)	
Unable to do activity	502 (3.73%)	119 (4.34%)	383 (3.58%)	
**Marital status**, ***n*** **(%)**				0.051
Living alone	4,957 (38.18%)	1,055 (39.83%)	3,902 (37.76%)	
Married or living with a partner	8,026 (61.82%)	1,594 (60.17%)	6,432 (62.24%)	
**Smoking**, ***n*** **(%)**				0.536
Never	6,928 (51.53%)	1,429 (52.06%)	5,499 (51.40%)	
Ever	6,516 (48.47%)	1,316 (47.94%)	5,200 (48.60%)	
**Diabetes**, ***n*** **(%)**				0.276
Yes	1,524 (11.34%)	295 (10.75%)	1,229 (11.49%)	
No	11,921 (88.66%)	2,450 (89.25%)	9,471 (88.51%)	
**Hypertension**, ***n*** **(%)**				0.288
Yes	4,255 (31.93%)	848 (31.09%)	3,407 (32.15%)	
No	9,071 (68.07%)	1,880 (68.91%)	7,191 (67.85%)	
**Stroke**, ***n*** **(%)**				0.065
Yes	471 (3.50%)	112 (4.08%)	359 (3.36%)	
No	12,974 (96.50%)	2,633 (95.92%)	10,341 (96.64%)	
**Coronary heart disease**, ***n*** **(%)**				0.002
Yes	684 (5.09%)	108 (3.93%)	576 (5.38%)	
No	12,761 (94.91%)	2,637 (96.07%)	10,124 (94.62%)	
Alcohol consumption (mean ± SD)	4.65 ± 34.39	4.16 ± 35.19	4.77 ± 34.18	<0.001
Family PIR (mean ± SD)	2.58 ± 1.54	2.27 ± 1.48	2.66 ± 1.55	<0.001
Vitamin C intake (mean ± SD), mg/day	98.91 ± 110.61	92.75 ± 118.97	100.49 ± 108.30	<0.001

BMI: body mass index; PIR: the ratio of income to poverty.

## 3 Results

### 3.1 Baseline characteristics of participants

Our research had 13,445 subjects, averaging 49.81 ± 19.06 years of age. 47.39% were male, and 52.61% were female. Severe headaches or migraines affected 2,745 people (32.09% males and 67.91% females). The participants' mean daily intake of vitamin C came in at 98.91 ± 110.61 mg, 92.75 ± 118.97 mg for those who had severe headaches or migraines, and 100.49 ± 108.30 mg for those who did not. In contrast to the group of participants who did not experience severe headaches or migraines, those who did experience these conditions tended to be female, younger, less educated, less non-Hispanic White, higher BMI, engage in less moderate physical activity, have a lower prevalence of coronary artery disease, drink less alcohol, make less money at home, and consume less vitamin C from diets (overall *P* < 0.05) (Table 1).

### 3.2 Correlation between transformed vitamin C consumption and severe headaches or migraines

The multivariate regression analyses of transformed vitamin C and severe headache or migraine are displayed in Table 2. Severe headaches or migraines were inversely correlated with transformed vitamin C consumption in model 1. Model 3 showed that this negative correlation remained stable (OR = 0.94; 95%CI: 0.91–0.98; *p* = 0.0007), showing that for every 1 mg/day increase in transformed vitamin C intake, the odds of a severe headache or migraine prevalence were 6% lower. To perform a sensitivity analysis, we also changed the continuous variable of transformed vitamin C consumption to a categorical variable (quartiles). In model 3, compared to quartile 1, the odds of suffering a severe headache or migraine were considerably lower in quartile 4 by 22%. However, the comparison of quartiles 1 and 2 (OR = 0.98; 95%CI: 0.86–1.10; *p* = 0.6956) and 3 (OR = 0.90; 95%CI: 0.79–1.02; *p* = 0.0885) did not reveal any statistically significant differences.

**Table 2:** Association between transformed vitamin C consumption and severe headaches or migraines.

**Exposure**	**Model 1 [OR (95%CI)]**	***P*-value**	**Model 2 [OR (95%CI)]**	***P-*value**	**Model 3 [OR (95%CI)]**	***P*-value**
Transformed Vitamin C	0.91 (0.88, 0.94)	<0.0001	0.91 (0.88, 0.94)	<0.0001	0.94 (0.91, 0.98)	0.0007
**Transformed Vitamin C**						
Quartile 1	Reference		Reference		Reference	
Quartile 2	0.90 (0.80, 1.01)	0.0623	0.91 (0.81, 1.03)	0.1363	0.98 (0.86, 1.10)	0.6956
Quartile 3	0.77 (0.68, 0.86)	<0.0001	0.82 (0.72, 0.92)	0.0011	0.90 (0.79, 1.02)	0.0885
Quartile 4	0.71 (0.63, 0.80)	<0.0001	0.71 (0.62, 0.80)	<0.0001	0.78 (0.69, 0.89)	0.0002
P for trend	0.87 (0.84, 0.91)	<0.0001	0.88 (0.84, 0.92)	<0.0001	0.92 (0.88, 0.96)	0.0002

Model 1: no covariates were adjusted. Model 2: adjusted were race, gender, and age. Model 3: adjustments were made for family PIR, age, gender, race, BMI, moderate activities, education level, married status, drinking, smoking, diabetes, hypertension, stroke, and coronary heart disease.

**Figure 2:**
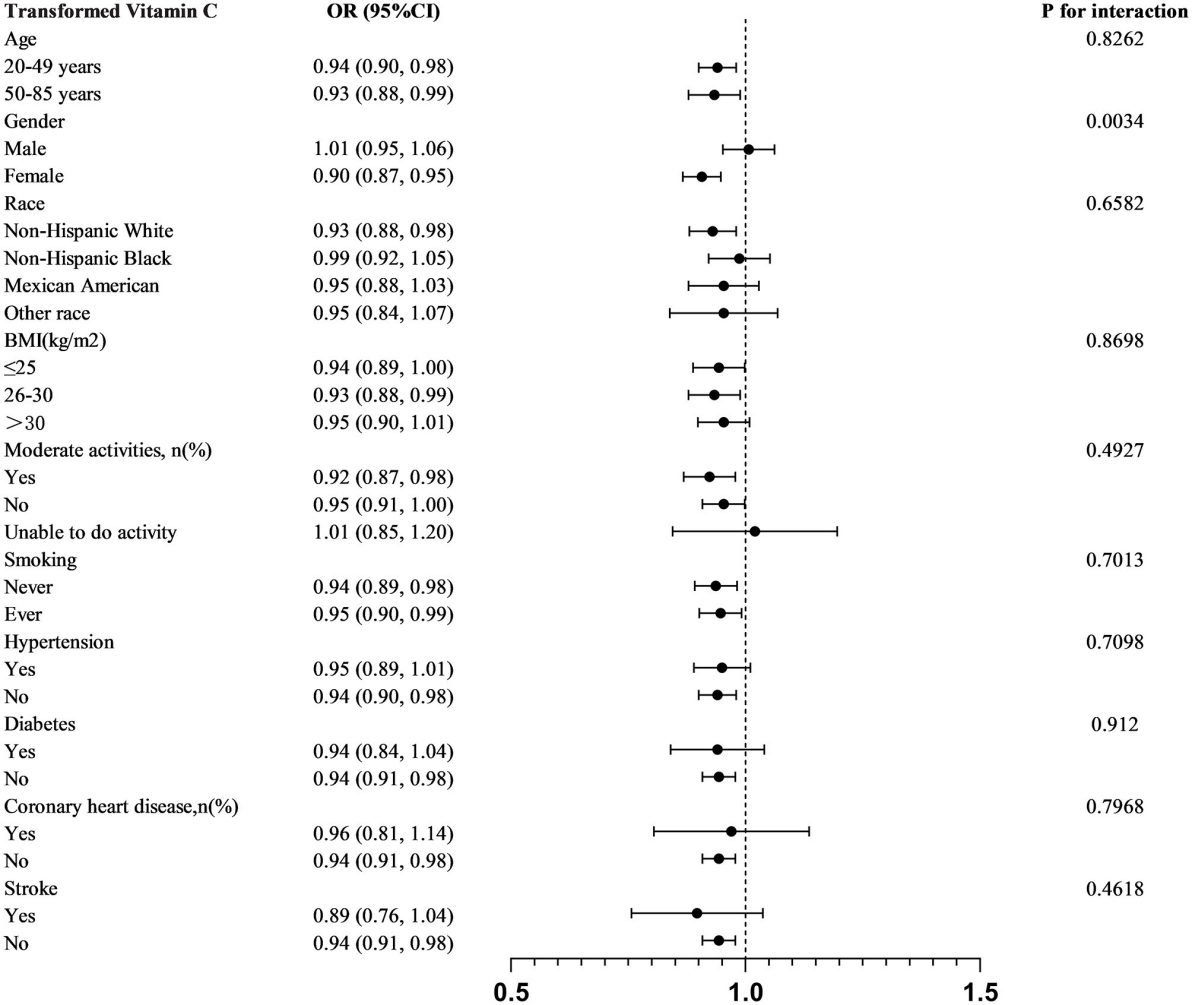
Subgroup analysis of transformed vitamin C consumption and severe headache or migraine.

We stratified analyses by age, sex, race, BMI, moderate physical activity, smoking, hypertension, diabetes mellitus, coronary heart disease, and stroke. In order to further investigate the variables impacting the connection between transformed vitamin C consumption and severe headaches or migraines, A statistically significant relationship was found in subgroup analyses stratified by gender between transformed vitamin C consumption and severe headache or migraine (*P* for interaction = 0.0034). Transformed vitamin C consumption was substantially and negatively correlated with severe headache or migraine in age, female, non-Hispanic white, BMI 26–30 kg/m^2^, moderately physically active (yes and no), smokers, non-hypertensive, non-diabetic, non-coronary heart disease, and non-stroke participants (*p* < 0.05) (Figure 2).

**Figure 3:**
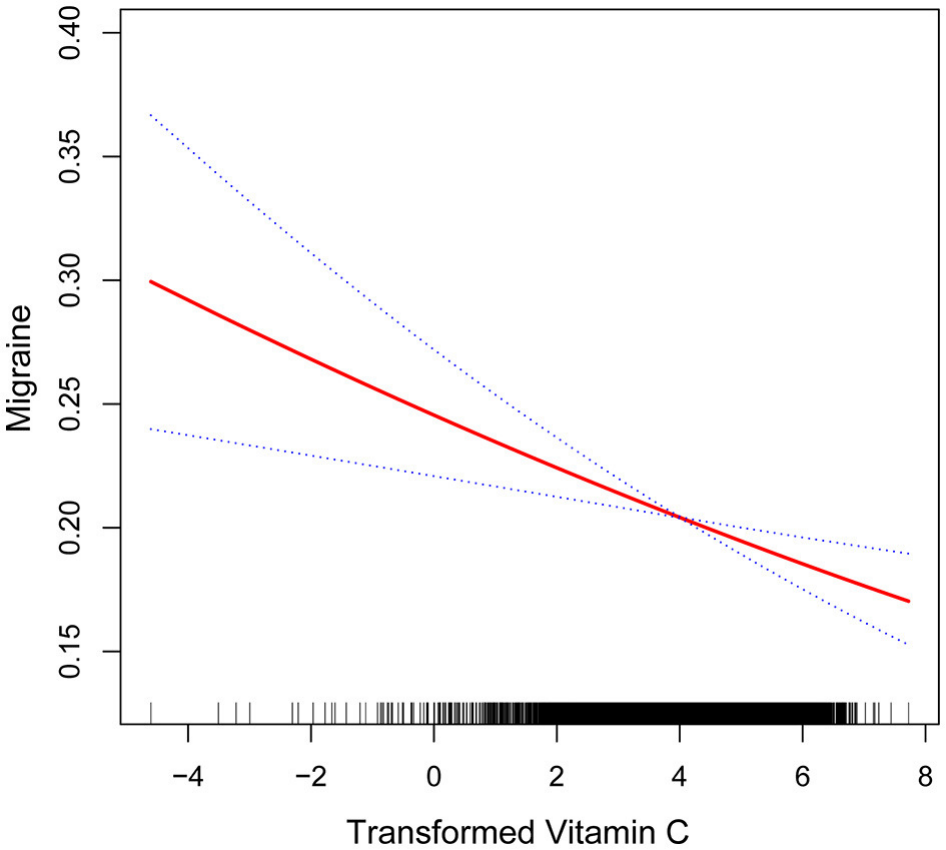
Non-linear association between transformed vitamin C consumption and severe headache or migraine. The smooth curve fit between the variables is shown by the solid red line. The 95% confidence interval derived from the fit is shown by blue bands.

**Figure 4:**
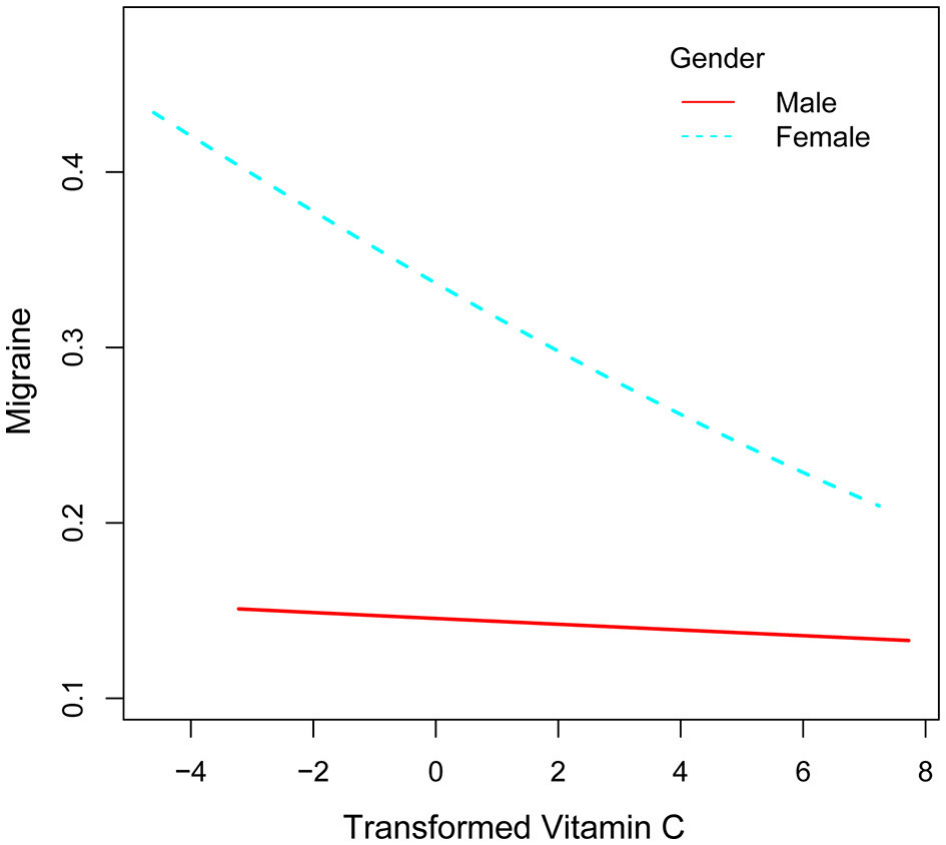
Correlation between converted vitamin C consumption and severe headache or migraine stratified by sex.

After that, the nonlinear association between transformed vitamin C consumption and severe headaches or migraines was shown using smoothed curve fitting (Figures 3, 4). The results showed an inverse relationship between these two variables (Figure 3). When stratified by gender, the negative correlation was highly significant among females (Figure 4). Age, gender, race, degree of education, BMI, physical activity, marital status, drinking, smoking, diabetes, hypertension, stroke, coronary heart disease, and household poverty-to-income ratio were all taken into account by the model.

## 4 Discussion

Vitamin C consumption was found to be negatively correlated with severe headaches or migraines in this cross-sectional investigation, which involved 13,445 people. Even after accounting for all confounders, this correlation is still significant. Furthermore, there was a substantial sex-based difference in the relationship between vitamin C consumption and severe headaches or migraines, with the female group's relationship being negative.

This was the first study, to our knowledge, to evaluate the connection between vitamin C consumption and migraines. According to a case report written by Bali et al. in 1978, a migraine patient experienced severe headaches whenever he skipped his daily 6g dose of vitamin C. This patient, with a 6-year history of migraines, stopped taking his medication after it proved effective and solely relied on a daily 6 g dose of vitamin C to manage his headaches, yielding remarkable results. When he took the placebo, he had terrible headaches, but on the days he took vitamin C, he had either no migraines or only mild migraines, according to the results of the double-blind study (34).

Chayasirisobhon (35) studied the effects of an antioxidant combination product on migraine patients. During this open-label study, patients took 10 capsules every morning for three consecutive months. Pine bark extract (120 mg), vitamin C (60 mg), and vitamin E (30 IU) were all included in each tablet. By the end of the therapy session, the patient's headache frequency and intensity had significantly decreased. Chayasirisobhon conducted an open-label trial to investigate the effects of vitamin C and pine bark extract on migraine symptoms. Patients received 1,200 mg of pine bark and 150 mg of vitamin C over the course of 3 months. For 58% of the individuals, this therapy significantly reduced headache frequency and intensity. Patients had long-term symptom relief and a more than 50% decrease with respect to both the frequency and intensity of subsequent migraine episodes after taking vitamin C and pine bark extract on a regular basis for a full year (36). A randomized controlled experiment was carried out by Visser et al. to look at the potential preventive impact of antioxidant drugs on migraine outcomes. A treatment group and a control group were randomly assigned to migraine sufferers for this trial. In the treatment group, patients received two capsules twice a day containing 300 mg of N-acetylcysteine, 125 IU of vitamin E, and 250 mg of vitamin C. Under the control group, patients received a capsule containing cellulose and food coloring. Patients in the therapy group had substantial decreases after 3 months in terms of mean monthly migraine episodes, headache ratings, usage of acute medication, and monthly migraine duration (37).

In a cross-sectional study, Peng et al. discovered that, in comparison to those without severe headaches or migraines, those with these conditions consumed less vitamin C, vitamin A, riboflavin, vitamin B6, and other nutrients (38). Using the validated Migraine Disability Assessment (MIDAS) questionnaire and the Visual Analog Scale (VAS) to measure migraine severity, Bahrampour et al. investigated the correlation between migraine duration and intensity and dietary nutritional patterns. The results showed that MIDAS and a first nutritional pattern in women—characterized by dietary calcium, magnesium, vitamin C, vitamin A, vitamin K, vitamin B6, and vitamin B2—correlated negatively (39). Our findings show a consistent trend with previous studies and provide a basis for further determining the efficacy of vitamin C intake in severe headaches or migraines.

It is unclear why vitamin C is associated with higher odds of severe headaches or migraines. However, the correlation between migraine and oxidative stress may support this negative association. A disruption in the equilibrium between pro-oxidants and antioxidants markers is termed oxidative stress. Disorders in cellular metabolic processes result in elevated levels of ROS and RNS, greater vulnerability to environmental oxidative substances, and a compromised antioxidant defense mechanism (9). In 2010, Alp et al. evaluated indicators of cellular redox state in migraineurs without aura for the first time, demonstrating variations in the oxidative stress index (OSI), total antioxidant status (TAS), and total oxidative status (TOS). In comparison with the control cohort, individuals diagnosed with migraine without premonitory symptoms had considerably higher TOS values and lower TAS values (40).

Cortical spreading depression is one of the main pathophysiological processes underlying migraine aura episodes (41), which is connected to the meninges, trigeminal sensory ganglia, and impacted cortex experiencing oxidative stress (42). Jiang et al. found that ROS, via alteration of TRPA1 ion channel activity, is essential in the pathogenesis of CSD. Furthermore, they demonstrated how antioxidants reduce the action of ROS, which lowers the risk of CSD *in vivo* (43). Calcitonin gene-related peptide (CGRP) constitutes another significant component in the pathogenesis of migraine. Using targeted cranial sampling from the external jugular vein, Goadsby et al. investigated the levels of numerous neuropeptides in migraine sufferers during bouts of headache. The finding was the first to demonstrate an elevation in calcitonin gene-related peptide (CGRP) within the extracranial circulation during migraine attacks. Since then, CGRP has garnered significant attention from researchers as a potential therapeutic target (44). Several *in vitro* findings have found that CGRP activates ganglionic glial cells to release nitric oxide (NO) (45), which encourages the release of CGRP, resulting in a ganglion's CGRP and NO positive feedback loop (46). Variations in plasma NO release and levels of vasoactive peptides, such as CGRP, can be brought on by CSD (47). Increased NO and CGRP levels cause harm to the respiratory chain of the mitochondria, reduce ATP synthesis, and generate substantial quantities of ROS. This causes oxidative stress and damaged neurons, which can trigger migraine headaches (48–51). NO and CGRP expression are also impacted by mitochondrial malfunction and excessive ROS generation, and these elements combine to cause migraine (42, 52–54).

Vitamin C acts as a powerful antioxidant, effectively slowing or stopping the oxidation process of diverse oxidizable substrates for instance proteins, lipids, carbohydrates, and nucleic acids (55). Its unique chemistry allows it to directly scavenge ROS and RNS, thus providing effective antioxidant protection against oxidative damage to other compounds (56). Furthermore, vitamin C has a secondary antioxidant effect that enhances the regeneration of other antioxidants, such as vitamin E (20). The reason why vitamin C is considered an ideal antioxidant stems from its ability to react with all physiologically relevant free radicals and oxidants. During free radical scavenging, ascorbic acid radicals converted from vitamin C can undergo further reactions or be reduced by specific enzyme systems, thus maintaining their continued antioxidant potency (57). A pivotal factor in maintaining homeostasis within the central nervous system lies in the comparatively abundant amounts of vitamin C located in certain brain areas. It is transported in the organism through diverse transporter proteins and exerts its antioxidant function within neurons, exhibiting significant neuroprotective effects, including reduction of neurotoxicity, prevention of oxidative stress, and resistance to neurodegenerative pathologies (58).

Damage to tissue or nerves can result in complex regional pain syndrome (CRPS), a severe neuroinflammatory condition affecting the limbs. According to epidemiological research by Mos et al., those with inflammatory bowel disease, asthmatics, and migraineurs had a higher chance of developing CRPS (59). Besse et al. suggested vitamin C as a prophylactic treatment for CRPS I because they found it to effectively mitigate the risk of secondary CRPS I after wrist and ankle fractures (60). Chen et al. found that 2 g of vitamin C before surgery provided some analgesia and could be used as an adjunct to reduce postoperative morphine dosage (moderate-level evidence). Following limb surgery, CRPS I was avoided with perioperative supplementation of 1 g of vitamin C daily for 50 days in a row (high-level evidence) (61). Neurogenic inflammation may be the common factor linking these seemingly unrelated disorders. Starr et al. found that Substance P (SP) and CGRP are two neuropeptides that are secreted when ROS are produced. This results in tissue damage and microvascular dysfunction in mice (62). Results of *in vitro* studies have shown elevated levels of SP, CGRP, and ROS in patients with CRPS and migraine (63, 64). Vitamin C, as an antioxidant, “scavenges” ROS produced during the early neurogenic inflammatory process of CRPS; therefore, it is logical to hypothesize that vitamin C modulates neurogenic inflammation and reduces ROS levels during migraine (65).

Our research has some advantages. First, as our data source, NHANES used a well-validated methodology for collecting dietary information that enhances credibility and ensures strong representation. Second, this study provides epidemiological evidence in a large, representative U.S. population indicating a substantial connection between dietary vitamin C consumption and severe headaches or migraines. Third, to ensure that the findings applied to a broad population, we adjusted for covariates associated with exposure and outcome. We performed stratified analyses of multiple subgroups to determine whether differences existed between subgroups. However, this study still has some limitations. Firstly, a 24-h recall was employed to gather the information regarding vitamin C consumption, which might introduce bias into the recall process. Secondly, rather than a clinical diagnosis made by experts, data on severe headaches or migraines were obtained using self-report questionnaires. Additionally, no information was available on the intensity, other symptoms, or subtypes of migraine that affected the participants' severe headaches or migraines. Furthermore, the possibility that the observed associations are the result of unaddressed confounders cannot be completely excluded, even though we have controlled for a number of confounding variables. Finally, we were unable to look at particular populations or different races since the survey only included adults in the United States and did not include special categories like minors. Consequently, more investigation is required to confirm if the findings are generally applicable.

## 5 Conclusion

Our data point to a strong negative connection between dietary vitamin C consumption and severe headaches or migraines. Vitamin C intake may have potential benefits for migraine sufferers in preventing or reducing migraine headaches. To corroborate the authors' findings, more large-scale prospective studies are required, as ours does not allow us to demonstrate causation.

## Data availability statement

The datasets presented in this study can be found in online repositories. The names of the repository/repositories and accession number(s) can be found in the article/supplementary material.

## Ethics statement

The studies involving humans were approved by National Center for Health Statistics (NCHS) Ethics Review Board (ERB) Approval. The studies were conducted in accordance with the local legislation and institutional requirements. The participants provided their written informed consent to participate in this study.

## Author contributions

YZ: Writing – review & editing, Writing – original draft, Software, Data curation. JJ: Writing – review & editing. CW: Writing – review & editing. CH: Writing – review & editing, Methodology.
